# Curcumin, Polydatin and Quercetin Synergistic Activity Protects from High-Glucose-Induced Inflammation and Oxidative Stress

**DOI:** 10.3390/antiox11061037

**Published:** 2022-05-24

**Authors:** Giulia Matacchione, Debora Valli, Andrea Silvestrini, Angelica Giuliani, Jacopo Sabbatinelli, Chiara Giordani, Sofia Coppari, Maria Rita Rippo, Maria Cristina Albertini, Fabiola Olivieri

**Affiliations:** 1 Department of Clinical and Molecular Sciences, DISCLIMO, Università Politecnica Delle Marche, 60126 Ancona, Italy; debora.valli@libero.it (D.V.); a.silvestrini@pm.univpm.it (A.S.); angelica.giuliani@staff.univpm.it (A.G.); j.sabbatinelli@staff.univpm.it (J.S.); c.giordani@pm.univpm.it (C.G.); m.r.rippo@staff.univpm.it (M.R.R.); f.olivieri@staff.univpm.it (F.O.); 2 Department of Biomolecular Sciences, University of Urbino Carlo Bo, 61029 Urbino, Italy; s.coppari3@campus.uniurb.it (S.C.); maria.albertini@uniurb.it (M.C.A.); 3 Center of Clinical Pathology and Innovative Therapy, IRCCS INRCA, 60127 Ancona, Italy

**Keywords:** hyperglycemia, natural compounds, T2DM, aging, oxidative stress

## Abstract

Chronic hyperglycemia, the diagnostic biomarker of Type 2 Diabetes Mellitus (T2DM), is a condition that fosters oxidative stress and proinflammatory signals, both involved in the promotion of cellular senescence. Senescent cells acquire a proinflammatory secretory phenotype, called SASP, exacerbating and perpetuating the detrimental effects of hyperglycemia. Bioactive compounds can exert antioxidant and anti-inflammatory properties. However, the synergistic anti-inflammatory and antioxidant effects of the most extensively investigated natural compounds have not been confirmed yet in senescent cells and in hyperglycemic conditions. Here, we exposed young and replicative senescent HUVEC (yHUVEC and sHUVEC) to a high-glucose (HG) condition (45 mM) and treated them with Polydatin (POL), Curcumin (CUR) and Quercetin (QRC), alone or in combination (MIX), to mirror the anti-inflammatory component OxiDef^TM^ contained in the novel nutraceutical Glicefen^TM^ (Mivell, Italy). In both yHUVEC and sHUVEC, the MIX significantly decreased the expression levels of inflammatory markers, such as MCP-1, IL-1β and IL-8, and ROS production. Importantly, in sHUVEC, a synergistic effect of the MIX was observed, suggesting its senomorphic activity. Moreover, the MIX was able to reduce the expression level of RAGE, a receptor involved in the activation of proinflammatory signaling. Overall, our data suggest that the consumption of nutraceuticals containing different natural compounds could be an adjuvant supplement to counteract proinflammatory and pro-oxidative signals induced by both hyperglycemic and senescence conditions.

## 1. Introduction

Chronic hyperglycemia, the diagnostic biomarker for Type 2 Diabetes Mellitus (T2DM), can foster a chronic low-grade proinflammatory status named inflammaging [1,2], nowadays recognized as the main risk factor for the development of the most common age-related diseases (ARDs) [3].

Aging is among the most effective risk factor of T2DM [4], as advanced age leads to the exacerbation of the inflammaging, promoted by the accumulation of senescent cells (SCs).

Cellular senescence is a process in which cells terminate to proliferate and acquire distinctive phenotypic and metabolic alterations, named senescence-associated secretory phenotype (SASP), with pro-inflammatory activity [5]. Indeed, in the last decade, the accumulation of SCs was identified in different tissues of T2DM patients and mouse models [6,7].

Though the main pathways involved in the proinflammatory activity of hyperglycemia were extensively investigated, the molecular mechanisms underpinning this activation are currently under investigation. A high glucose level can stimulate the production of reactive oxygen (ROS) and nitrogen species [8]. Hyperglycemia is also related to an increased production of advanced glycation end products (AGE), derived by non-enzymatic protein glycation, as observed in the serum/plasma of patients affected by T2DM [9]. AGEs can stimulate immune and non-immune cells, i.e., endothelial cells, to release a plethora of pro-inflammatory cytokines, through their binding to the cellular isoform of AGE receptor (RAGE). Human endothelial cells can be stimulated by AGEs and ROS, which activate NF-kB transcription factor and increase the synthesis and releases of inflammatory mediators such as tumor necrosis factor-alpha (TNF-α), interleukin-1β (IL-1β) and chemokines, such as monocyte/macrophage chemotactic protein-1 (MCP-1) and interleukin-8 (IL-8) [10]. Several studies have reported that a hyperglycemic condition markedly stimulate monocyte adhesion, vascular permeability, ROS formation and NF-kB activation in human endothelial cells [11,12,13,14,15]. Hyperglycemia is therefore considered an important causative factor in the development of micro- and macrovascular complications in patients affected by T2DM [4,16].

Among the compounds playing antioxidant and anti-inflammatory activities, polyphenols are the most extensively investigated both in animal models and in human clinical trials [17,18,19]. Clinical and epidemiological evidence strongly suggests that diets rich in polyphenols can reduce the risk of development of several age-related chronic diseases [20]. In this framework, the number of studies related to the usefulness of consuming compounds derived from natural sources has increased, with the purpose to create differentiated products with high added value. Here, we investigated the properties of three of the most extensively investigated natural compounds, i.e., Curcumin (CUR), Polydatin (POL) and Quercetin (QRC), alone and in combination (MIX), to mirror the anti-inflammatory component, named OxiDef^TM^, of the novel nutraceutical Glicefen^TM^ (Mivell, Italy).

Curcumin, a yellow-orange lipophilic compound from *Curcuma longa*, and Polydatin, a precursor of Resveratrol, are recognized powerful modulators of inflammation [21,22]. Clinical trials and in vitro studies have reported the role of Curcumin in reducing the pro-inflammatory status occurring in T2DM patients and its complications [23,24]. Polydatin exhibits many biological functions, among which the inhibition of adipose tissue inflammation in high-fat-fed mice [25] and the improvement of diabetic conditions in streptozotocin-induced diabetic rats [26,27] as well as of DSS-induced colitis in mice [28].

Quercetin is a very promising bioactive compound, with anti-inflammatory and antioxidant effects in high-glucose-treated endothelial and mesangial cells [29,30,31], with anti-senescence effects [32] and with pro-health effects in animal models [33,34].

However, although each of these phytochemicals has been deeply studied alone, their synergistic anti-inflammatory activity has not been extensively investigated, in particular in human endothelial cells in conditions of senescence and hyperglycemia, about which no studies have been published [35,36]. In this study, we aimed to evaluate a possible synergistic activity of Curcumin, Polydatin and Quercetin bioactive compounds, by testing their anti-inflammatory and antioxidant activities in high-glucose-treated young and senescent human endothelial cells (HUVECs).

## 2. Materials and Methods

### 2.1. HUVEC

Human umbilical vein endothelial cells (HUVEC) are primary pooled cells that were obtained from Clonetics (Lonza, Switzerland). HUVEC were cultured in endothelial growth medium (EGM-2, Lonza, Switzerland), composed of endothelial basal medium (EBM-2, Lonza, Switzerland) and the SingleQuot Bullet Kit (Lonza, Switzerland). The cells were seeded at a density of 5000/cm^2^ in T75 flasks (Corning Costar, Sigma Aldrich, St. Louis, MO, USA).

### 2.2. Characterization of Young and Senescent HUVEC Cells

Replicative senescence (RS) was reached by subsequent replicative passages (measured as cumulative population doubling, cPD). cPD was calculated as the sum of PD changes, calculated by the formula: (log_10_ (F) − log_10_ (I))/log_10_ [2], where F is the number of cells at the end of a passage, and I is the number of seeded cells. HUVECs were classified as young or senescent based on cPD, senescence-associated (SA)-β-Galactosidase activity and p16^ink^⁴^a^ expression. SA-β-Gal activity was detected by using a Senescence Detection Kit (BioVision Inc., Milpitas, CA, USA), following the manufacturer’s instructions.

### 2.3. Cell Viability Assay

We tested cell viability by using the MTT (3-(4,5-dimethylthiazol-2-yl)-2,5-diphenyltetrazolium bromide) assay. The cells were grown for 24 h and 72 h in 24-well plates at a density of 5000 cells/cm^2^ before treatments with different doses of the natural compounds as well as in high glucose conditions (25–35–45 mM). Briefly, the MTT (1 mg/mL) solution was added, and the cells were incubated for 4 h; the obtained product, a formazan salt, was solubilized in dimethyl sulfoxide (DMSO) and measured by a microplate reader (MPT Reader, Invitrogen, Milano, Italy) at the optical density of 540 nm.

Cell viability was calculated according to the equation (T/C) × 100%, where T and C represent the mean optical density of the treated group and of the control group, respectively.

### 2.4. High-Glucose Treatments

To achieve hyperglycemic conditions (HG), HUVEC were treated with D-Glucose for 24 and 72 h. Three different concentrations (25 mM, 35 mM and 45 mM) were tested. HUVECs were grown in EGM-2 medium (D-Glucose 5 mM) as a control (NG).

### 2.5. Natural Compound Treatments

Curcumin (218580100) was purchased from Acros organic. Polydatin (P1878) and Quercetin (P0042) were purchased from TCI. All compounds were dissolved in DMSO at a 0.1% final concentration of DMSO in all the solutions. Based on the results of the viability assay, the cells were treated with 1 µM Curcumin, 10 µM Polydatin and 0.5 µM Quercetin, alone or in combination (MIX, 1 µM Curcumin, 10 µM Polydatin and 0.5 µM Quercetin). The MIX resembles the anti-inflammatory component OxiDef^TM^ of the nutraceutical Glicefen^TM^ (patent number:102021000032909). The treatments with the single compounds and the MIX consisted in a 2 h pretreatment, followed by a 24 h treatment for young HUVEC in HG conditions and a 72 h treatment for senescent HUVEC in HG conditions. The synergistic effect was calculated by measuring the anti-inflammatory effect (to be intended as a reduction of the expression of markers of inflammation with respect to their expression in inflamed HUVEC) of each single compound and of the MIX. We considered the MIX as synergistic if the effect of the MIX was greater than the sum of the effects of each compound acting separately.

### 2.6. RNA Isolation, mRNA and Mature miRNAs Expression by RT-qPCR

Total RNA was isolated by using the Norgen Biotek Kit (Thorold, ON, Canada), according to the manufacturer’s instructions and stored at −80 °C until use. mRNA and miRNA expression was determined as previously described [37]. RT-qPCR analysis were standardized with RNU48 for miRNA expression and with GAPDH and β-actin for mRNA expression.

### 2.7. Western Blot Analysis

RIPA buffer (0.1% SDS, 150 mM NaCl, 1.0% Triton X-100, 10 mM Tris, 5 mM EDTA pH 8.0) with a protease and phosphatase inhibitor cocktail (Roche Applied Science, Indianapolis, IN, USA) was used to obtain the cell lysates. The Bradford assay was used to evaluate the protein concentration in each sample. Proteins (25 μg) were analyzed by SDS-PAGE, and then transferred to a nitrocellulose membrane (Bio-Rad, Hercules, CA, USA). A blocking buffer (Bio-Rad, Hercules, CA, USA) was used to block the membrane that was then incubated overnight with primary antibodies.

Mouse anti-phospho-p38 (Cell Signaling), rabbit anti-phospho-NF-kB (Cell Signaling), mouse anti-PCNA (Cell Signaling) and mouse anti-*β*-actin (Cell Signaling) were used as primary antibodies.

We used secondary horseradish peroxidase-conjugated antibodies (anti-mouse or anti-rabbit) (The Jackson laboratory, Bar Harbor, ME, USA). A Uvitec Imager (UVItec, Cambridge, UK) was used to visualize the protein bands by using the Clarity ECL chemiluminescence substrate (Bio-Rad) that were then quantified using ImageJ software. Each measure was normalized with respect to *β*-actin.

### 2.8. ELISA Assay

Conditioned medium from the cell cultures was collected at the end of each incubation, centrifuged at 14.000 × RPM for 20 min and stored at −80 °C until use. IL-8 ELISA Kit (Invitrogen, Waltham, MA, USA) was used to measure the concentration of IL-8 released in the medium, according to the manufacturer’s instructions.

### 2.9. DCFDA Assay

The 2′,7′ –dichlorofluorescin diacetate (DCFDA) assay is based on the quantification of 2′, 7′ –dichlorofluorescin (DCF) within cells, which indicates the presence of ROS. DCFDA enters the cells and is deacetylated by cellular esterases to a non-fluorescent compound, which is later oxidized by ROS into DCF. DCF is a highly fluorescent compound with maximum excitation/emission spectra at 495/529 nm. yHUVEC and sHUVEC were seeded in a dark, clear-bottom 96-well plate at 5000 cells/cm^2^ in 100 µL of growth medium and allowed to grow for 24 h at 37 °C, 5% CO_2_, 95% humidity. The treatments with the single compounds and the MIX consisted in a 2 h pretreatment, followed by a 4 h treatment for young HUVEC in HG conditions and a 48 h treatment for senescent HUVEC in HG conditions. Forty-five minutes prior to the completion of the treatment, 100 µL of 40 µM DCFDA diluted in the same media used for treatment (containing the experimental compounds) was added to each well. Fluorescence intensity was measured at the excitation wavelength of 485 nm and the emission wavelength of 535 nm on a Biotek plate reader.

### 2.10. Statistical Analysis

Data are shown as mean ± SD or frequency (%) of three independent biological replicates. Data of RT-qPCR, ELISA and densitometry were analyzed by using the paired sample T test. Data analysis was performed using IBM SPSS Statistics for Windows, version 25 (IBM Corp., Armonk, NY, USA). Statistical significance was defined as a two-tailed *p*-value < 0.05.

## 3. Results and Discussion

### 3.1. High-Glucose Treatment in yHUVEC and sHUVEC Induces Pro-Inflammatory Responses

**Figure 1:**
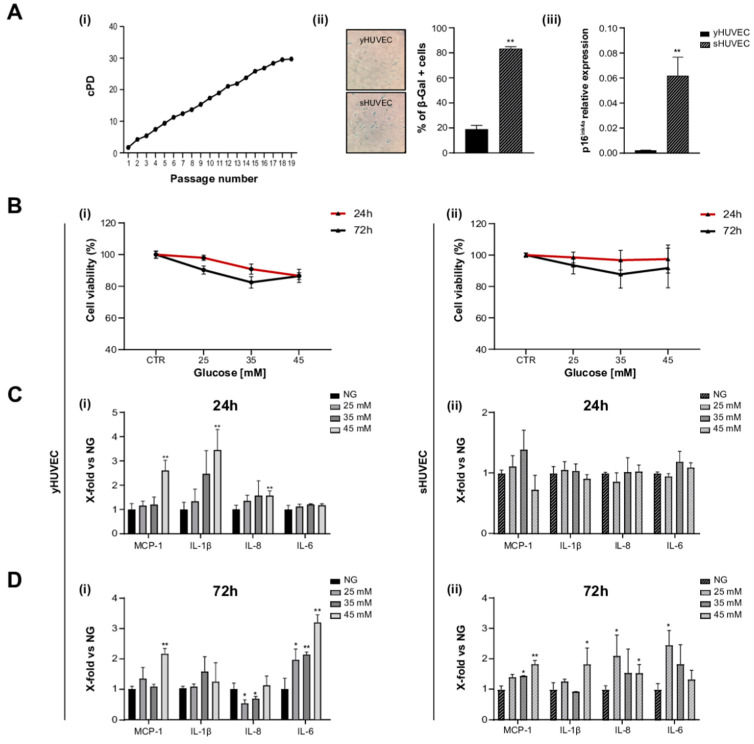
High glucose concentration induced inflammation in yHUVEC and sHUVEC. Characterization of sHUVEC by a cumulative population doubling curve (**i**), β-Gal activity (**ii**) and p16^ink4a^ expression level (**iii**) (**A**). Effect of high-glucose treatments on cell viability. yHUVEC (**i**, left-hand side panel) and sHUVEC (**ii**, right-hand side panel) cells were treated with 25, 35 and 45 mM glucose for 24 and 72 h. Cell viability was determined by the MTT assay (**B**). Relative mRNA expression of MCP-1, IL-1β, IL-8 and IL-6 in yHUVEC (**i**, left-hand side panel) and sHUVEC (**ii**, right-hand side panel) in cells upon treatment with 25, 35 and 45 mM of glucose for 24 h (**C**) and 72 h (**D**). Results are expressed as mean ± SD of three independent biological replicates. Asterisks (*) indicate significance versus NG; * *p* < 0.05, ** *p* < 0.01. NG, normal glucose; HG, high glucose.

HUVEC were considered (i) young (yHUVEC) when they were at replicative passage <6, (SA)-β-Gal activity was <20%, and p16^ink^⁴^a^ expression was significantly lower compared to its level in senescent HUVEC, (ii) senescent (sHUVEC) when at a replicative passage >15, (SA)-β- < Gal activity was >60%, and p16^ink^⁴^a^ expression was significantly higher than its level in yHUVEC (Figure 1A(i–iii)).

yHUVEC and sHUVEC were exposed to different concentrations of glucose (D-Glucose) (25, 35 and 45 mM) to identify potential side effects (decreased cell viability) and proinflammatory stimulation, after 24 and 72 h of treatment.

In all these experimental conditions, cell viability did not decrease under 70% compared to control cells both for yHUVEC and for sHUVEC cells (Figure 1B(i, ii)). When the inflammatory response to different D-Glucose concentrations was tested, pro-inflammatory cytokines (IL-1β and IL-6) and chemokines (MCP-1 and IL-8) were differently modulated in yHUVEC compared to sHUVEC. yHUVEC showed a significant up-regulation of MCP-1, IL-1β and IL-8 after 24 h of treatment with 45 mM D-Glucose (Figure 1C(i)). The exposure of yHUVEC to all the tested concentrations of D-Glucose was associated with a significant up-regulation of IL-6, but only at 72 h (Figure 1D(i)). When yHUVEC were exposed to 45 mM D-Glucose, MCP-1 was significantly up-regulated at both 24 and 72 h (Figure 1C(i),D(i)).

sHUVEC, did not show any modulation of the pro-inflammatory markers after 24 h (Figure 1C(ii)), but the levels of MCP-1, IL-1β and IL-8 were significantly increased after 72 h of exposure to 45 mM D-Glucose (Figure 1D(ii)).

These results allowed us to specifically select the D-Glucose treatment conditions, i.e., glucose concentration and time of exposure, effectively associated with an inflammatory response in yHUVEC and sHUVEC. All subsequent experiments were performed with 45 mM D-Glucose, treating young (24 h) and senescent cells (72 h) for different time.

It is not surprising that young and senescent cells are characterized by a different mean time of inflammatory responses when subjected to stressful stimuli. As an increased transcriptional activity of pro-inflammatory molecules is a key feature of senescent cells, it is reasonable to assume that young cells can rapidly increase the synthesis and release of proinflammatory compounds in response to harmful stimuli, whereas senescent cells with SASPs require a longer treatment with stressogenic stimuli to be able to observe an intensive proinflammatory response [38].

### 3.2. Polydatin, Curcumin and Quercetin Effects on HUVECs Viability

**Figure 2:**
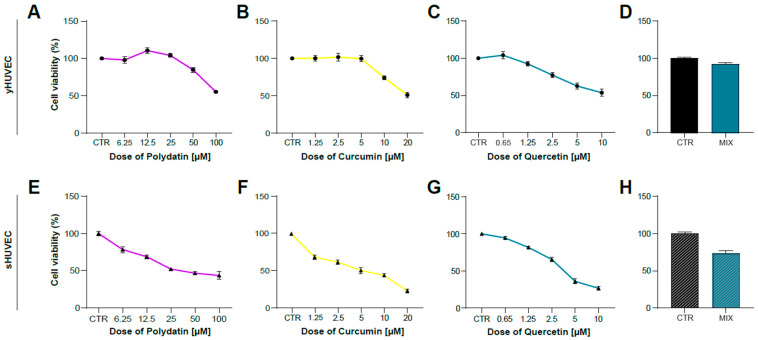
Dose–response curve of yHUVEC and sHUVEC to Polydatin, Curcumin, Quercetin and combination treatment. yHUVEC (**A**–**D**) and sHUVEC (**E**–**H**) were treated with different concentrations of Polydatin (from 6.25 µM to 100 µM), Curcumin (from 1.25 µM to 20 µM), Quercetin (from 0.65 µM to 10 µM), MIX or with DMSO alone as a control for the indicated times. The MTT assay was used to assess the effect of the treatments on cell viability. The results indicate the percentage of cell viability normalized to the viability of DMSO-treated cells (CTR) and presented as mean value ±SD from three independent biological replicates.

After identifying the appropriate experimental conditions to mimic in vitro the inflammatory activation of endothelial cells induced by hyperglycemia, we tested the anti-inflammatory properties of Polydatin (POL), Curcumin (CUR) and Quercetin (QRC) natural compounds, either alone or mixed together (MIX). This experimental approach was applied to verify their potential synergistic effects. We previously reported that the combination of some natural compounds was able to improve their anti-inflammatory properties [37]. We firstly evaluated cell viability of yHUVEC (Figure 2A–D) and sHUVEC (Figure 2E–H) treated for 24 h and 72 h, respectively, with different concentrations of natural compounds alone. The concentrations to be used for subsequent experiments were selected based on cell viability > 70% compared to that of control HUVEC and resulted to be 10 μM POL, 1 μM CUR, 0.5 μM QRC, whether used individually or in combination (MIX).

### 3.3. The Combined Natural Compounds Exert Anti-Inflammatory Activity on yHUVEC

**Figure 3:**
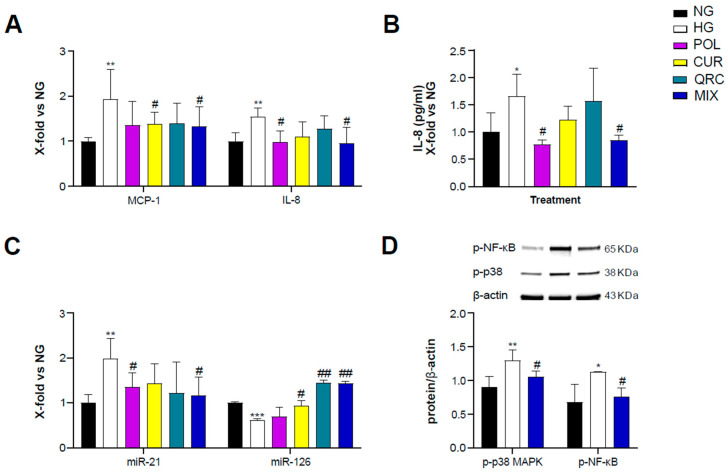
Effect of each compound following exposure to high glucose in yHUVEC. The cells were treated for 24 h with a single compound or with their combination. Relative mRNA expression of MCP-1 and IL-8 (**A**), concentration (pg/mL) of IL-8 in the culture medium (**B**), expression levels of miR-21 and miR-126 (**C**), representative western blot analysis showing p-p38 MAPK and p-NF-kB expression in yHUVEC treated with NG, HG or the MIX (**D**). β -actin levels were used as a control. The bands were quantified by ImageJ. All data are reported as fold change vs. untreated young HUVEC. The results are expressed as mean ±SD from three independent biological replicates. In all panels, asterisks (*) indicate significance versus NG; (#) indicates significance versus HG; one symbol, *p* < 0.05; two symbols, *p* < 0.01; three symbols, *p* < 0.001. NG, normal glucose; HG, high glucose; POL, polydatin; CUR, curcumin; QRC, quercetin; p-p38, phosphorylated-p38; p-NF-kB, phosphorylated-NF-kB; β -act, β -actin.

To evaluate the anti-inflammatory activity of POL, CUR and QRC, yHUVEC were pre-treated for 2 h with 10 µM POL, 1 µM CUR, 0.5 µM QRC or the MIX (10 µM POL, 1 µM CUR and 0.5 µM QRC) and then treated for 24 h with D-Glucose (45 mM). We observed that the MIX significantly decreased the expression of MCP-1 and IL-8 but we did not observe a synergistic effect (Table 1), whereas CUR and POL alone significantly down-regulated MCP-1 and IL-8, respectively, compared to control yHUVEC (Figure 3A). These results were confirmed by the quantification of IL-8 chemokine release in the culture medium. High-glucose conditions were able to induce a significant up-regulation of IL-8 release, which was significantly reduced by the treatments with POL and the MIX (Figure 3B). IL-1β mRNA expression was not modulated by any of the three substances, either alone or in combination (Figure S1A).

Since specific microRNAs such as miR-21 and miR-126 and miR-146a, belonging to the group of inflamma-miRs, are able to modulate inflammatory pathways and endothelial cell health [39,40,41,42,43] and are involved in the modulation of transcriptional programs related to the performance of endothelial cells [44], here we also analyzed their expression/modulation under HG and natural compounds treatment. miR-21 appeared significantly up-regulated in HG-exposed yHUVEC compared to control cells. Importantly, POL and MIX treatments were both associated with a significant decrease of miR-21 levels, suggesting a reduction of the inflammatory response. A significant down-regulation of miR-126 was observed in HG-treated yHUVEC, and this phenomenon was significantly reverted by treatment with CUR, QRC and the MIX (Figure 3C), suggesting that these compounds can ameliorate the function of endothelial cells. In contrast, we observed increased miR-146a expression in yHUVEC exposed to HG, but the single substances and the MIX were ineffective in reducing it (Figure S1B).

Finally, we aimed to identify the pathways involved in the anti-inflammatory activity of the MIX. Among various intracellular proteins that can modulate inflammation, we selected p38 mitogen-activated protein kinases (MAPK), since it is one of the major players during inflammatory responses [45]. Indeed, p38 MAPK regulates the transcriptional activity of NF-kB by controlling the phosphorylation of the p65 subunit in a IKKγ-independent manner [46]. We found that the activation of p38 MAPK and the phosphorylation of NF-kB (p65 subunit) were increased in yHUVEC in high-glucose conditions compared to the control and significantly reduced by the MIX treatment (Figure 3D). This result suggests that the MIX could significantly restrain NF-kB activation.

### 3.4. Synergistic Anti-Inflammatory Effect of Natural Compounds on sHUVEC

**Figure 4:**
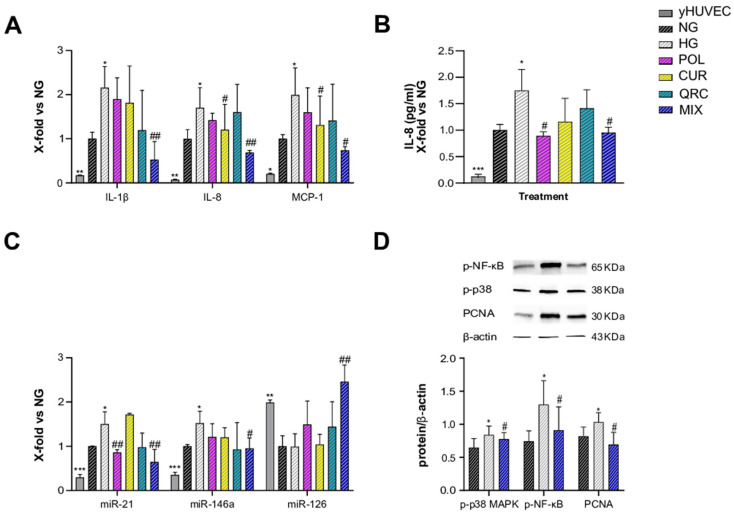
Effect of each compound following exposure to high glucose in sHUVEC. Cells were treated for 72 h with a single compound or with their combination. Relative mRNA expression of IL-1β, IL-8 and MCP-1 (**A**), concentration (pg/mL) of IL-8 in the culture medium (**B**), miRNA expression levels of miR-21, miR-146a and miR-126 (**C**), representative western blot analysis showing p-p38 MAPK, p-NF-kB and PCNA expression in sHUVEC cells treated with NG, HG or the MIX (**D**). β -actin levels were used as a control. The bands were quantified by ImageJ. All data are reported as fold change vs. untreated senescent HUVECs. The results are expressed as mean ±SD from three independent biological replicates. In all panels, asterisks (*) indicate significance versus NG; (#) indicates significance versus HG; one symbol, *p* < 0.05; two symbols, *p* < 0.01; three symbols *p* < 0.001. NG, normal glucose; HG, high glucose; POL, polydatin; CUR, curcumin; QRC, quercetin; p-p38, phosphorylated-p38; p-NF-kB, phosphorylated-NF-kB; PCNA, proliferating cell nuclear antigen; β -act, β -actin.

As T2DM is an age-related disease, and aging is characterized by an increased burden of senescent cells, we aimed to address the anti-inflammatory effects of POL, CUR and QRC on senescent endothelial cells. sHUVEC were pre-treated for 2 h with all the natural compounds (10 µM POL, 1 µM CUR and 0.5 µM QRC or with their combination (MIX)), before adding glucose (45 mM D-Glucose) for 72 h exposure. First, sHUVEC were compared to yHUVEC. The expression of the main pro-inflammatory markers (IL-1β, IL-8, MCP-1, miR-21 and miR-146) confirmed that sHUVEC were characterized by an elevated inflammatory status at basal level (Figure 4A–C). Then, we noted that only the MIX was able to significantly reduce the expression of IL-1β, whereas CUR alone and the MIX significantly down-regulated IL-8 and MCP-1 expression compared to control cells (Figure 4A). Notably, we achieved a synergistic effect of the three natural compounds in reducing IL-1β and IL-8 expression (Table 2), as their combined action was greater than the sum of each individual compound’s action [47].

Another interesting result is that POL e MIX treatments were able to significantly reduce the release of IL-8, which was increased in the medium of sHUVEC exposed to hyperglycemia (Figure 4B). These data suggest a senomorphic activity of these compounds, that is, a suppression of SASP effects without cell death. Senomorphic treatments provide an alternative pharmacological approach to target cellular senescence, since they suppress the detrimental effects of SASP components secreted by senescent cells without causing cell death.

When the expression levels of miR-21, miR-146a and miR-126 were evaluated in sHUVEC, miR-21 showed a significant reduction in cells exposed to HG treated with POL, and, importantly, an even more relevant decrease was obtained with MIX treatment. A significant down-regulation was observed also for miR-146a in sHUVEC treated with the MIX. MiR-21 and miR-146a were previously identified as miRNAs able to restrain the activation of NF-kB, and therefore a reduction of their levels is indicative of an anti-inflammatory response [48]. Of note, even if we did not find any significant difference in the expression levels of miR-126 between sHUVEC cultured in high-glucose conditions and control cells cultured in normoglycemic medium, we obtained a synergistic effect of the MIX on the up-regulation of miR-126 expression compared to each tested compound (Figure 4C).

Lastly, increased activation of p38-MAPK and phosphorylation of p65-NF-kB were confirmed in sHUVEC exposed to HG, both modulated by the treatment with the MIX (Figure 4D). Besides, we also assessed the modulation of Proliferating Cell Nuclear Antigen (PCNA), as this molecule is involved in DNA damage repair [49]. In HG-sHUVEC. the level of PCNA was significant increased compared to control cells, indicating that a high glucose level can induce DNA damage. Of note, the treatment with the MIX significantly down-regulated the PCNA level (Figure 4D), thus suggesting a role of the natural compounds also in reducing DNA damage.

### 3.5. Antioxidants Activity of POL, CUR and QRC on yHUVEC and sHUVEC

**Figure 5:**
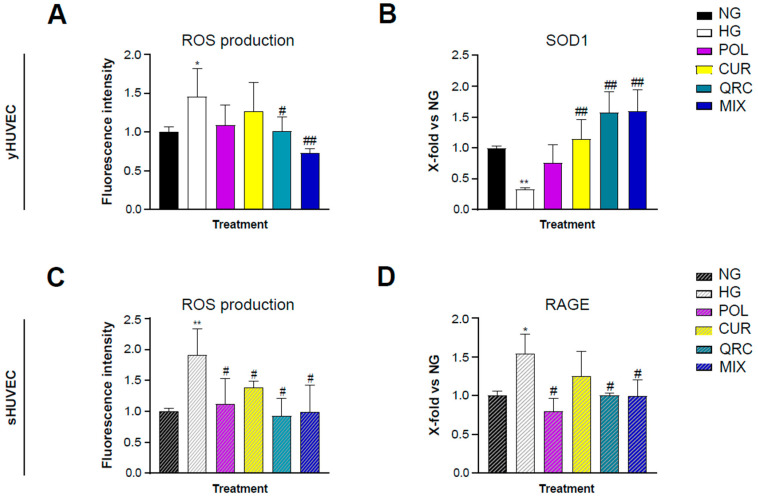
Effect of each natural compound on HG-induced ROS production in HUVEC. The cells were treated with a single compound or with their combination (MIX). Reactive oxygen species concentration was measured using the DCFDA assay in yHUVEC (**A**) and sHUVEC (**C**) cells. Relative mRNA expression of SOD1 (**B**) and RAGE (**D**) in yHUVEC and sHUVEC, respectively. All data are reported as fold change vs. untreated HUVEC cells. Results are expressed as mean ±SD from three independent biological replicates. In all panels, asterisks (*) indicate significance versus NG; (#) indicates significance versus HG; one symbol, *p* < 0.05; two symbols, *p* < 0.01. NG, normal glucose; HG, high glucose; POL, polydatin; CUR, curcumin; QRC, quercetin.

It is well established that hyperglycemia leads to an increased production of reactive oxygen species (ROS) through several mechanisms [50]. Therapeutic strategies involving antioxidant natural molecules have become popular in the last decades. As expected, our experimental conditions showed a significant increase of ROS production in HG-exposed yHUVEC (Figure 5A) and sHUVEC (Figure 5C). Interestingly, only the treatment with QRC, and even more with MIX, induced a significant reduction of ROS levels in yHUVEC compared to the cells in HG conditions (Figure 5A). In sHUVEC, each of the three compounds, either alone or in combination, had a significant effect in reducing the production of ROS (Figure 5C).

The expression of superoxide dismutase type 1 (SOD1) was also analyzed, since it is one of the main enzymes involved in ROS removal and conversion. SOD1 expression was significantly down-regulated in yHUVEC in HG compared to NG conditions. The treatment with CUR, QRC and MIX was associated with a substantial increase of SOD1 expression (Figure 5B). In sHUVEC, we did not find any modulation of SOD1 expression (data not shown). As in HUVECs the acquisition of the senescence phenotype is related to oxidative stress [51], we hypothesized that high-glucose conditions might not be able to induce a significative modification of SOD1 activity.

Thus, we evaluated the expression of RAGE, the receptor for AGE, whose formation is accelerated in high-glucose conditions and can promote activation of the NF-kB pathway and increased oxidative stress through its binding to RAGE [52]. Interestingly, we found that sHUVEC exposed to HG conditions expressed a significative higher level of RAGE compared to control cells, which was significantly reduced at the mRNA level by POL, QRC and the MIX (Figure 5D).

## 4. Conclusions

Both in in vitro and in vivo models, a hyperglycemic status was demonstrated to deregulate the functionality of the endothelium, promoting the development of vascular complications and dysfunction [53]. Hyperglycemia is a diagnostic parameter for T2DM, a complex metabolic disorder characterized by glucidic and lipidic metabolism imbalance. At the cellular and molecular levels, a high-glucose condition is associated with increased ROS, responsible for vascular inflammation, nitric oxide (NO) synthesis inhibition, insulin resistance and protein and macromolecule glycation inducing the formation of AGEs.

Increasing evidence strongly suggests that nutritional supplement intake can contribute to improving endothelial dysfunction in T2DM patients.

In our study, we observed a synergistic effect of a specific combination of Polydatin, Curcumin and Quercetin (OxiDef^TM^, Mivell), only in sHUVEC, in reducing the expression levels of IL-8 and IL-1β, two main SASP components, supporting the view that some natural compounds can act synergistically as “senomorphic”, suppressing the most relevant markers of SASP and thus reducing the exacerbation and the spreading of SASP, that can fuel a systemic inflammatory chronic condition.

Overall, the most relevant and innovative results reported in our manuscript are the evaluation of the combination of two pro-inflammatory conditions: cellular senescence and hyperglycemia. This model mirrors the conditions that can be both present in vivo in diabetic patients, especially in those older and with poorer glycemic control, and therefore at higher risk of developing severe complications.

Our in vitro results strongly suggest testing OxiDef^TM^ as an adjuvant treatment in T2DM patients.

## Figures and Tables

**Table 1:** Effect of POL, CUR, QRC and MIX on gene expression versus expression after HG treatment in yHUVEC.

yHUVEC	MCP-1		IL-8		miR-126	
	Effect vs. HG (%)	Synergistic Effect *	Effect vs. HG (%)	Synergistic Effect *	Effect vs. HG (%)	Synergistic Effect *
POL	58	N	57	N	8	N
CUR	55		44		33	
QRC	54		27		84	
MIX	61		58		82	

N = None. * The effect of the three compounds (POL, CUR and QRC) taken together (MIX) is greater than the sum of their separate effect at the same doses.

**Table 2:** Effect of POL, CUR, QRC and the MIX on gene expression versus expression after HG treatment in sHUVEC.

sHUVEC	IL-1B		IL-8		miR-126	
	Effect vs. HG (%)	Synergistic Effect *	Effect vs. HG (%)	Synergistic Effect *	Effect vs. HG (%)	Synergistic Effect
POL	26	Y	28	Y	50	Y
CUR	34		50		5	
QRC	96		10		45	
MIX	163		102		145	

Y = Yes. * The effect of the three compounds (POL, CUR and QRC) taken together (MIX) is greater than the sum of their separate effect at the same doses.

## Data Availability

Data are contained within the article or supplementary material.

## Supplementary Materials

The following supporting information can be downloaded at: https://www.mdpi.com/article/10.3390/antiox11061037/s1.
