# Supplementary material for: Curcumin, Polydatin and Quercetin Synergistic Activity Protects from High-Glucose-Induced Inflammation and Oxidative Stress
Source: Antioxidants (Basel). 2022 May 24;11(6):1037. doi: 10.3390/antiox11061037 (PMC9220232; doi:10.3390/antiox11061037)
Supplement: Supplementary file 1 [file antioxidants-11-01037-s001.zip › antioxidants-1726092-supplementary.pdf]

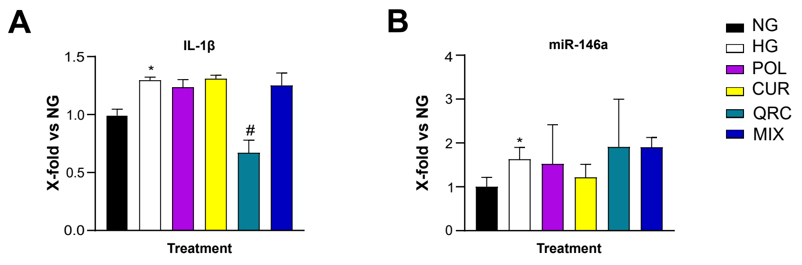

Figure S1. Effect of compound treatment following exposure to high glucose in yHUVEC cells.

Cells were treated for 24h with a single compound or with their combination. Relative mRNA expression of IL-1β (A) and relative miRNA expression of miR-146a (B). Results are expressed as mean ±SD from three independent biological replicates. Asterisks (*) indicate significance versus NG; * $p<0.05$, ** $p<0.01$. NG, normal glucose; HG, high glucose.
